# Premature termination of eating disorder treatment - a qualitative study of therapist perspectives

**DOI:** 10.1186/s40337-025-01268-0

**Published:** 2025-05-01

**Authors:** Oda Ulven, Signe Hjelen Stige, Yngvild Sørebø Danielsen

**Affiliations:** The University of Bergen, Bergen, Norway

**Keywords:** Eating disorders, Anorexia nervosa, Bulimia nervosa, Binge eating disorder, Dropout, Drop-out, Premature termination of treatment, Therapist perspective, Reflexive thematic analysis

## Abstract

**Background:**

High dropout rates are a problem in eating disorder treatment and the knowledge about how therapists who work in this field understand and intervene to reduce drop out is scarce. The aim of this study was to explore how therapists understand and work to prevent dropout during eating disorder treatment.

**Methods:**

Five focus group interviews with a total number of 17 therapists were conducted. The therapists all worked in specialized mental health care and had different occupational backgrounds, including psychologists, specialist nurses, and doctors. All of them reported receiving training in CBT-E. Transcripts were analysed using a reflexive thematic analysis.

**Results:**

Our analysis resulted in the development of three main themes: (1) Accepting dropout; (2) Balancing resources; and (3) Recognizing the demands of treatment.

**Conclusions:**

Our findings suggest that preparation for treatment is conceptualized by therapists as something quite separate from treatment itself and as crucial for preventing dropout and providing good treatment results. Interestingly, interventions and processes during treatment received much less focus in the discussions among therapists during the interviews.

**Trial registration:**

This project has been approved by Regional Committees for Medical and Health Research Ethics in Norway (REK), REK-number 55,304.

## Abstract (plain language summary)

High dropout rates are a problem in eating disorder treatment and the knowledge about how therapists who work in this field understand and intervene to reduce drop out is scarce. The aim of this study was to explore how therapists understand and work to prevent dropout during eating disorder treatment using focus group interviews. Our analysis resulted in three main themes: (1) Accepting dropout; (2) Balancing resources; and (3) Recognizing the demands of treatment. The findings suggest that preparation for treatment is conceptualized by therapists as something quite separate from treatment itself and as crucial for preventing dropout and providing good treatment results. Interestingly, interventions and processes during treatment received much less focus in the discussions among therapists during the interviews.

## Background

EDs are associated with high mortality, reduced quality of life, significant distress, as well as compromised physical and mental health [1]. AN is the deadliest mental disorder, with up to 5 times the mortality compared to the general population. In addition to high individual costs, EDs are linked to substantial societal expenses. In Norway, the total societal costs associated with EDs are estimated to be 26 billion Norwegian kroner, equivalent to more than 2.3 billion euros, in 2021 [2].

Although effective ways to treat ED exist [3], high dropout rates represent a key challenge when providing treatment for ED. Dropout from psychotherapy can be defined as when a patient “discontinues an intervention prematurely, before recovering from the problems that led him or her to seek out treatment and/or before completing the intervention’s specified protocol” [4, p.193]. Dropout rates in eating disorder treatment vary, ranging from 20-51% for inpatients and 29–73% for outpatients [5]. Even before specialized outpatient treatment starts, the estimated dropout rate is between 13 and 32% [6]. It is noteworthy that in one study only 48% of individuals recommended for treatment choose to enrol, with 36% subsequently dropping out [7]. The consequences of leaving treatment prematurely can be severe. Studies have found that dropout leads to poorer treatment outcomes and prognosis [8–10]. Patients who discontinue treatment are less likely to recover on their own [11] and this group often faces a poorer long-term outcome, leading to re-referral to treatment when the illness has become more severe and long lasting [12, 13]. A recent study found that 32.5% of patients drop out of treatment prematurely, and that premature dropout might be a factor contributing to low rates of remission [14].

Understanding and addressing the factors contributing to dropout is of importance for improving treatment outcomes and the long-term recovery prospects of individuals with EDs. Unfortunately, identifying consistent predictive factors for dropout across studies remains challenging due to methodological variations and differing definitions of dropout [5, 13, 15–17]. There is no established definition of dropout. The definition varies widely, from patients leaving treatment against professional advice to therapists discharging patients for non-compliance or failure to meet treatment goals [16]. Limitations like small sample sizes, limited study replications, and a lack of diversity among patient populations also make it difficult to draw conclusions regarding the nature of and motivations for dropout during treatment [18]. Despite efforts to investigate dropout, the existing literature provides inconclusive findings, with a predominant focus on quantitative research. These studies have mainly studied how pre-treatment demography, co-morbidity, personality or aspects of the ED predicts later dropout, with none of these factors found to reliably be associated with dropout [19]. Vinchenzo and colleagues [18] conclude that additional qualitative studies should be done to clarify meanings of dropout as different expectations held by patients and by therapeutic teams could impact the therapeutic alliance and therefore dropout rates.

In general, finding consistent predictors for dropout in psychotherapy treatment has been challenging and the literature is contradictive due to a variety of methodological problems [20]. Reis and Brown suggest that reducing differences between therapists’ and patients’ perspectives on the therapeutic process could lead to fewer psychotherapy dropouts. Variables such as clients’ intentions and expectations and client–therapist interactions have for example been found to be stronger associated with treatment dropout than simple client and therapist variables [21]. This also seems to be the case in eating disorder treatment [22]. This indicates the need for qualitative research exploring in-depth both patient and caregiver perspectives and experiences with processes leading to prematurely ending treatment.

To address this gap in the literature, we plan to conduct a larger project that explores both client and therapist perspectives. The main aim of the project is to produce knowledge that can help us understand more about dropout and difficulties with engaging in treatment for eating disorders. The study will consist of several sub-studies to explore the perspectives of the key actors in the treatment of eating disorders through qualitative interviews, a survey and register data. In this article, we will focus on the therapist perspective.

To the best of our knowledge, there has been limited research on the therapist’s perspective regarding dropout in ED treatment. We argue that insight into how clinicians perceive, prevent and handle dropout during treatment is crucial to enhance our understanding of the phenomenon, and will contribute to more knowledge about how treatment systems operate in response to dropouts. Their insights can highlight practical challenges and potential solutions that may not be evident from quantitative data alone. Therapists’ attitudes and perceptions of dropout can influence the admission process in eating disorder treatment. If therapists view dropout as a common and expected outcome, they may be more vigilant in identifying at-risk patients early on and implementing strategies to prevent dropout. Conversely, if dropout is seen as a minor issue, it might not receive the necessary attention, potentially leading to higher dropout rates.

Exploring the therapists’ perspectives on treatment dropout can shed light on systemic barriers within the healthcare system that lead to higher dropout rates. In addition, clinicians have a perception of the patient’s experience, symptoms, and challenges, therefore therapists offer valuable insights into why so many patients discontinue treatment and could suggest strategies to support them in remaining engaged. They might be in a position to influence clients’ experiences during treatment in order to prevent dropout. By recognizing and addressing the therapist perspectives, it might be possible to enhance our ability to prevent dropout to a greater degree and improve overall treatment outcomes.

## Methods

### Aim, study setting and design

The aim of this sub-study was to explore how therapists understand and work to prevent dropout during eating disorder treatment. Since we were interested in exploring and gaining an in-depth understanding of the topic, we chose to use a qualitative design that would allow for a deep exploration of the phenomenon and provide detailed insights into participants’ experiences and perspectives.

This study was conducted within the context of outpatient specialized mental health care clinics in Western Norway. The clinics are part of the public healthcare system in Norway, where you have the right to necessary medical care from the specialist health services without insurance cover. When prioritizing care, healthcare services have to assess whether the patient can be expected to benefit from the care and the expected resource use is reasonably proportionate to the expected benefit [23]. According to the Norwegian Directorate of Health the expected benefits of healthcare should be assessed based on whether evidence-based practices indicate that the healthcare can extend the patient’s lifespan or improve their quality of life. Patients have the right to be re-referred for treatment also in cases where they previously have discontinued treatment as long as they meet the criteria for the right to necessary healthcare. The Norwegian national guidelines for treatment of EDs recommend that psychotherapy targeted at EDs is used for treating adult patients with AN, BN and BED [24]. Enhanced cognitive-behavioural therapy for eating disorders (CBT-E) is suggested as treatment for adults with BN, whereas there is no specific treatment proposed for treating AN or BED. Nevertheless, most clinicians that work in specialized mental health care are offered training in CBT-E.

To collect data, we chose to use focus group interviews since we were interested in information from therapists working together in teams. A focus group interview (FGI) is a qualitative research method that involves a small group of participants discussing a specific topic or issue under the guidance of a moderator [25]. The aim is to explore personal experiences, beliefs, perceptions, and attitudes of the participants through group discussions.

### Data collection and materials

#### Participants and recruitment

We established cooperation with seven different clinics where clinicians working with treatment of EDs in an outpatient setting were invited to participate in FGIs. In five of the clinics, therapists accepted this opportunity. Three of these clinics were outpatient clinics and two of them had established specialized teams treating EDs. Two of the clinics specialized exclusively in the treatment of EDs. One of the specialized clinics work exclusively with outpatients whereby all the therapists participated in the FGI. The other specialized clinic had both outpatients and inpatients, whereby only the therapists working with outpatients participated in the FGI. The therapists were invited through their workplace, and therefore, we do not have a complete overview of all who received the information. However, the impression was that most therapists were present in all teams. Prior to the interviews the participants were given information about the project and the background for the study in a one-hour session. All participants signed a written consent prior to the FGIs.

In total, our study involved the participation of 17 therapists, representing diverse professional backgrounds including psychologists, specialist nurses, and medical doctors. 16 of the 17 participants were female. Experience levels among the therapists varied, but most had extensive backgrounds in working with individuals with EDs. Table 1 shows an overview over the participants’ education, age, and years of experience with ED. All of them reported receiving training in CBT-E and that they primarily used this approach in their clinical practice. In all teams, CBT-E was identified as the preferred treatment method. However, in 3 of the FGIs they reported that alternative approaches were considered if CBT-E had been previously attempted without success or if it was deemed unsuitable. Seven therapists reported having additional training, with six having further education in emotion-focused therapy (EFT) and one using a psychodynamic approach when CBT-E was not appropriate. One team had access to a psychomotor physiotherapist, an art therapist, and a nutritionist. These professionals could either assist in treatment or manage patients independently, but were frequently integrated into treatment plans, primarily in combination with CBT-E.

**Table 1:** Overview of participants’ education, age, and years of experience with ED

Education	*n*	Age in years	*n*	Years of experience with ED	*n*
Clinical Psychologist	4	20–29	1	< 5	4
Clinical Psychologist with Specialization	6	30–39	5	> 5	4
Psychiatric Nurse	6	40–49	4	> 10	2
Psychiatrist	1	50–59	5	> 15	4
		60–69	2	> 20	3

#### Interview guide

The interview guide was developed by the researchers based on experiences from previous, similar studies. Additionally, we established a collaboration with representatives from the user organization Counselling on Eating Disorders (ROS) based on the research questions in the project. ROS is offering a low-threshold service for everyone who directly or indirectly has been affected by EDs or struggle with issues related to food and body. The employees at ROS have previous personal experience with EDs. The representatives from ROS were invited to provide input on the questions and topics they considered important to discuss.

Open-ended questions were developed to allow the informants to discuss their own experiences without being too heavily guided. The aim was to explore how therapists recognize a patient who is at risk of dropping out of treatment for EDs and how they understand the phenomenon of dropout from ED treatment. The interview guide also contained questions about how the therapist would intervene to prevent dropout. We remained attentive to the potential need to adjust the interview guide during the process but determined that no adjustments were necessary. See Appendix 1 for the complete interview guide.

#### Data collection

In this study design the researchers chose to use two moderators, one primarily being the facilitator and the other one assisting by operating the recording equipment, asking follow-up questions, and writing down more detailed information. We conducted five FGIs; one session was held for each group lasting from one to two hours. SHS led the first FGI, while OU was the moderator. OU led the remaining FGIs. SHS moderated one of them, while YSD was the moderator for two of the others. The last FGI was conducted without a moderator due to illness.

The interviews were scheduled to take place during the therapists’ regular working hours at their respective clinics. Using this approach, we wanted to create a conducive and familiar atmosphere for open discussion [28]. Each focus group consisted of three to four therapists, primarily working as a cohesive team. There was one exception, as one clinic did not have established a specialized team for this purpose, however the therapists in this group stated that they often consulted each other but in a more informal way. The team leader participated in the interviews, but the formal leader of the clinics did not. As the teams were small, the team leader was considered an important part of the team. The team leaders were also working as therapists. Our overall impression is that the discussions flowed smoothly in most groups, with all participants actively involved. The co-facilitator monitored this engagement closely. However, there was one group where a participant was less involved, which is important to respect in terms of voluntary participation. Interviews were audio recorded and transcribed verbatim by the first author.

### Data analysis

Data analysis followed a reflexive thematic analysis (RTA) approach as outlined by Braun and Clarke [26, 27]. RTA is a theoretically flexible approach to analysing qualitative data. In this analysis a phenomenological-hermeneutic approach was employed to explore the lived experiences and meaning making of the participants [28]. This method combines phenomenology, which focuses on the structure of experience, with hermeneutics, which emphasizes the interpretation of meaning. The aim was to gain a deeper understanding of the participants’ perspectives and the meanings they ascribe to their experiences.

Initially, OU familiarized herself with the data material through the transcription of interviews and repeated reading of the transcripts (phase 1 of RTA; 26, 27). Interesting aspects of the data were identified through reading of the material. In this process all researchers read through the transcripts and noted down initial ideas of particularly rich parts of the data material and potential analytical foci.

The research team then met to discuss and decide analytical focus. We decided on a split analytical focus: therapists’ conceptualization of dropout and therapists’ interventions to prevent dropout. Data were therefore analysed twice, first, to understand how therapists conceptualized dropout, and second, to identify the interventions they used to reduce dropout.

OU coded the transcripts. N-Vivo R14.23.2 software [29] was employed as a technical tool to assist in the coding of the data. In N-Vivo the material was reread systematically sentence by sentence, and with each analytical focus in mind, meaningful excerpts were labelled and organized into codes (phase 2 of RTA; 26, 27). Each interview was coded separately. Existing codes were used if they were appropriate, and new codes were created if necessary. All the codes were eventually included in a collective analysis.

Following line-by-line coding with the split analytical focus, the research team met to start abstracting patterns of meanings from the coded material (phase 3–5 of RTA; 26, 27). Different themes and possible thematical organization were considered, tested and reviewed. This was done separately for each analytical focus.

Various tentative solutions for thematic structure were discussed iteratively among the research team to ensure the robustness of the identified themes. Themes were named and defined. After repeated discussion, we decided on three main themes that cut across analytical foci. We felt this structure best captured the key meaning dimensions in relation to the analytical foci and research questions.

Initially, we wanted to explore therapists’ understanding of dropout and strategies they used to prevent dropout. To our surprise, the therapists reported that they had few dropouts once they started treatment. Instead, they reported that a number of patients decline treatment during assessment or what the therapists referred to as the “preparation” phase for treatment. This is an interesting discovery in itself, but using the word “dropout” might be problematic since the therapy had not “officially” started yet. Maybe a different terminology to describe this phenomenon would have been more suitable such as “failure to engage in therapy” or “premature termination of treatment”. However, the preparation phase could consist of several sessions and continue for weeks, and sometimes even months. Consequently, we felt that it would be odd not to use the word “dropout” to describe this phenomenon. We also felt that “dropout” captured a broader understanding of the phenomenon and would be more suitable since later dropout in treatment was also discussed.

The writing of themes involved choosing codes and extract of data (phase 6 of RTA; 26, 27). In this process there is always a possibility that valuable insights may be overlooked or excluded from this final product. Nevertheless, we hope that we have manged to capture the essence of the participants meanings as well as a deeper understanding of the topic. After writing the results we reviewed the entire data set to validate that the final thematic structure reflected the data material.

### Reflexivity statement

The primary researcher, OU, is a newly graduated female psychologist with a keen interest in working with individuals with EDs clinically. At the beginning of the project, OU had limited clinical experience and little previous practice with this patient group. She had some knowledge of research in the field as she had written a master’s thesis about dropout in treatment. When the researchers conducted and transcribed the FGIs, OU still had not started working with EDs. However, while OU started coding and writing the article, she also started working at a specialized unit for EDs and thus gained experience and knowledge. OU has also received training in CBT-E after the transcribing of the data material, during the analysis. These experiences have allowed her understanding of the interviews and their content to evolve throughout the process. Initially, she was surprised by the level of responsibility placed on the patients. However, through CBT-E training, she came to understand that making the patient actively choosing to engage in treatment is a key component of the treatment model. Working with this project, however, OU has tried to analyse and write the article without being theoretically driven.

YSD has extensive experience with both research and treatment of EDs. She is an Associate Professor at the University of Bergen (UIB) and has worked as a clinical psychologist treating EDs for several years with training in both cognitive behavioural therapy and emotion focused therapy.

SHS is a Professor at UIB and a clinical psychologist. She is an experienced qualitative researcher, primarily from qualitative psychotherapy research and the field of complex trauma. She has prior experience working with the therapist perspective in psychotherapy research but is not particularly experienced in the field of EDs. The research team used her outsider position to help with reflexive processes during the research project. However, it’s essential to acknowledge that her position could have left her disadvantaged in relation to engagement with the phenomena being studied, as she has limited experience with clinical work with EDs. This, for example, became an issue for discussion following the FGIs she moderated, as she was perplexed by some of the therapist perspectives expressed during the interviews.

### Ethics

This project has been approved by Regional Committees for Medical and Health Research Ethics in Norway (REK), REK-number 55,304. All participants have signed a written consent.

Participation in the project was voluntary, but since the interviews were conducted in the workplace, there is a possibility that some participants may have felt an obligation to participate. To prevent people from feeling obliged to participate, we provided information about the project in advance where we emphasized that participation was voluntary, and all participants had to sign a written consent prior to participating in the interviews.

All participants had training and experience in a specific method (CBT-E), which could influence their responses from a method-specific perspective rather than personal preferences. It is possible that the participants might have felt that speaking from personal experiences divergent from method-specific approaches could be perceived as inappropriate or a lack of knowledge.

After the interviews, we held an open conversation about the process, and our overall impression was that the participants had a positive experience in participating.

## Results

Our analysis of the participants’ experiences resulted in the development of three main themes: (1) Accepting dropout; (2) Balancing resources; and (3) Recognizing the demands of treatment.

### Accepting dropout

Therapists consistently emphasized that the decision to engage in treatment should ultimately be the patient’s decision. They stressed the importance of providing patients with comprehensive information about the treatment process beforehand, enabling them to make an informed choice about whether to choose treatment. Regardless of whether patients accepted or declined treatment, therapists highlighted the importance of ensuring that the choices made by patients were respected.

Several therapists shared their experiences, emphasizing that it was crucial not to attempt to persuade or “push” patients into treatment. They viewed dropout as a phenomenon that, for some individuals, could be a necessary step that might lead them to return to treatment at a later, more suitable time.

We try to be aware of providing the patient with the feeling of actually having a real choice. That they are the ones that must choose treatment and commit to it. We work with patients aged 18 and up and they often have a long history of treatment in Child and Adolescent Mental Health Services (CAMHS). There they did not feel that they had a real choice. They often found the alliance with the therapist difficult and that their experienced difficulties were not addressed. I think this may be related to their level of maturity. There is a transformation that occurs during those years when they are in CAMHS. I think that patients making their own choice about treatment and taking on an active role is really significant. My colleagues often remind me that I must not get too active on the patients’ behalf, but rather support them to take ownership of their own treatment.

Respondent K, clinical psychologist, interview 3

In two of the groups, experiences were shared that being too active and pushy in persuading patients to choose treatment could paradoxically result in reduced engagement in patients or total dropout of treatment.

But it’s like patients and their families and others around them think, “You (the therapist) must do something, you must do something,” all the time, but as soon as we become action-oriented and try to do something, “Now we’re going to help you,” and show empathy, “I’m going to help fix this,” then we lose patients.

Respondent G, clinical psychologist, interview 2

Several therapists explained that they therefore tried to “lean back” or “sit on their hands” to avoid being too overly persuasive with the patient. They wanted to let the patient choose treatment without feeling coerced. There was a shared understanding that this approach could promote engagement and motivation for treatment in patients. This could lead to the use of interventions like this therapist described:

(…) be completely laid back about the decision. And it’s like when they start wanting treatment, we don’t jump on to it, almost like “I don’t know if you’ll get treatment,” to make them become even more engaged– “I want it, I’m going to have it”.

Respondent G, clinical psychologist, interview 2

Interestingly, several therapists expressed the perspective that they did not categorize patients declining therapy or discontinuing treatment as “dropouts” as long as there had been an open conversation about it, and they could understand the reasons behind the patients’ choices, even when these patients still had an active ED.

But I also often feel that when it comes to dropout, I refuse to call it that, it doesn’t feel like dropout; it feels like a choice and a decision that is good and right.

Respondent M, nurse, interview 4

It was a recurring topic in the group discussions that several therapists did not necessarily perceive dropout from treatment as something negative.

Some should indeed discontinue and return later; it’s not as if the goal is to have zero dropouts, in a way.

Respondent E, clinical psychologist, interview 2

Another therapist described:

I discussed one of my patients that is heading toward dropout in a team meeting that we had yesterday, and we pretty much agreed that this was a positive dropout. Well, yeah, where it’s appropriate.

Respondent A, clinical psychologist, interview 1

Many explained that they viewed treatment from a longer-term perspective, emphasizing the importance of patients feeling respected and having a positive experience with the process. Describing dropout as some sort of necessary evil.

I think dropping out is a lot of things. We talk as if it’s a negative thing when a patient declines treatment. But, well, we don’t have data that confirms that. What we do have is years of experience where patients who decline in a proper manner often come back. I don’t know how many of them do, but we remember only those who return. I believe that if I do thorough work before we agree to start a change project, the most crucial thing for me is that the patient says yes or no on a solid basis. And when they say no, I often think it was unfortunate, but I could very well consider it a positive outcome. I also think that the patient will hopefully come back because of the way we arrived at the decision when the patient said no. Quite often, patients return and say, “Now, I’ve been thinking differently; now, I want to try something else.” So, I care about that aspect because we want to help.

Respondent G, clinical psychologist, interview 2

Several other therapists shared experiences where patients returned at a later stage when they felt ready.

I have a strong belief underlying it all that it’s ultimately their choice. Regardless of their choices, my life doesn’t get any better, I also think that they make those choices for themselves, and quite often, those are the best choices because they are the ones dealing with it, and who am I to say what they should choose? But I also want to assume that they can come back because, in a way, I feel that they have started a more long-term process. So, I don’t mean that ending the treatment is necessarily wrong or anything. I think it’s great that we realized that there wasn’t a basis for continuing right now. They may feel that they are concluding in a good way so that they can withdraw if they need to. I feel that’s important [Yes, it’s important, indeed], yes, it always is.

Respondent M, nurse, interview 4

### Balancing resources

In nearly all the FGIs, the therapists emphasized the significance of thoroughly assessing potential factors that might inhibit the treatment process. The therapists characterized ED treatment as demanding and challenging for the patients, especially when patients were concurrently dealing with other life challenges. They described a reluctance to initiate treatment unless they were confident in the patient’s ability to see it through. Being ready for treatment was usually conceptualized as being willing to engage in behavioural changes such as eating regularly and gaining weight.

In the discussion, it became clear that the therapists believed that the chances of successful recovery were enhanced when the patient’s life circumstances were optimal, allowing them to prioritize and fully engage in treatment. If the therapists perceived the patient’s current life circumstances as excessively overwhelming, the therapists would refrain from offering patients treatment for their ED. One of the reasons for withholding treatment, as discussed in some of the group sessions, can be referred to as the concept of a “one shot.” This was often described as a singular or limited opportunity for getting well, underscoring the importance of not squandering that chance. It seemed like the therapists felt they carried responsibility, feeling compelled to ensure they do not initiate treatment prematurely and thereby deprive the patient of the opportunity to recover. Therefore, if the therapists concluded that a patient’s life situation was overly demanding, despite their willingness to engage in treatment, therapists may choose not to initiate treatment at that time. In their discussions, one of the therapists referred to the concept of a “golden ticket”:

There are so many things that we need to comprehensively assess together with the patient and discuss a bit, explaining how we approach it. This is because when we offer our treatment, it is focused and intense. It’s like a high dose over a relatively short period, which means it’s very demanding for the patients. However, we also explain to them that there’s a very good chance of getting better if everything is well in place in their life. So, in a way, we reserve that ‘golden ticket’ for people to start quickly when we collectively feel that the timing is as good as it can be. Then there are those everyday things, like ongoing family conflicts, ongoing stressors, deaths, financial problems, difficult work situations that they find challenging to step away from or prioritize down a bit. It could be exam periods, travel, weddings.

Respondent L, medical Doctor, interview 3

Many of the therapists also discussed the notion that it is better for patients not to enter treatment than to engage in a therapeutic process that does not yield positive results. There seemed to be a consensus that participating in treatment that does not progress can be demotivating and potentially more harmful than beneficial. Some even described it as “unethical” to continue treatment if there was not any progress.

I feel very strongly that it’s unethical to ask them to continue if I don’t expect them to make it to the finish line because there are some obstacles. And it’s crucial to have a dialogue about it. If those obstacles exist, I think it’s important [to address it], and often they can follow, you know, they see it. They see that, yes, and they also understand that as a treatment institution, we can’t keep them here and pretend they’re receiving treatment, right?

Respondent M, nurse, interview 4

Not making any progress in treatment was described as one of the most common reasons for ending the treatment pre-maturely, often initiated by the therapists.

I think we, in a way, focus a lot on an eating disorder not being the kind of illness where just talking is a good solution. Then nothing happens. Its behaviour change that needs to occur, especially in these most severe cases. So, if that doesn’t happen, we don’t “pretend to give treatment,” so to speak. We talk about “what’s happening” and “we can’t continue like this,” right? So, it’s either a change in their living situation or “come back later” or [Mm]… yes, that kind of dropout is mostly what I have in mind.

Respondent L, medical Doctor, interview 3

Some of the therapists pointed out that due to limited resources in the specialized healthcare system they had to prioritize patients they believed were able to benefit from treatment.

Sometimes they need a long time, more than a few months, and the question is how long we can retain patients in ambivalence exploration here in specialized healthcare, and unfortunately, it’s not as long as some need. Sometimes we start a bit too quickly, and other times we have to say, ‘I’m sorry,’ and they become upset and don’t quite understand, but we still have to say, “we cannot offer treatment” (…).

Respondent N, clinical psychologist, interview 4

One of the therapists mentions spending even less time on motivating the patient for treatment compared to previous practices because of the overwhelming number of patients within the healthcare system. She notes a change in the therapists’ approach, indicating a tendency to promptly clarify and assess situational factors and clinical condition in newly referred patients. Thus, leading to a speedy determination of appropriateness for treatment.

There are so many patients in the system [“Yes, there are”], and such an enormous demand, and it’s easy to become somewhat cynical. I find that I’ve become much quicker in clarifying things with newly referred patients, much faster in concluding that “okay, but it’s not right. Now you know what we have to offer, welcome back.” I spend less time motivating them than we did before because now we see the whole system.

Respondent A, clinical psychologist, interview 1

The participants described an upfront and thorough assessment process as a strategy to prevent dropout, rather than a cause for dropping out, even though the selective nature of offering treatment meant some patients would not get the chance to initiate therapy. None of the therapists used the word “dropout” to describe patients leaving or discontinuing treatment in the assessment process.

My experience is at least that when I see patients starting treatment and then encountering difficulties in following through, it’s often the case. In those moments, I often think, “Darn, we should have spent more time on those topics before we began.” That’s my main experience, thinking, “Darn, we were a bit too quick on that topic.” So, taking our time and perhaps getting the whole picture first.

Respondent N, clinical psychologist, interview 4

### Recognizing the demands of treatment

In the interviews, the therapists talked about the importance of understanding the cost patients experience in treatment. They highlight examples of how the treatment can simply be too demanding for the patients. Further, they underscored that some patients may not fully grasp the demanding nature of the treatment, and the intensity of the emotional and psychological challenges it requires. Despite the therapists’ efforts to provide comprehensive information about the treatment beforehand and about potential difficulties, the experience of treatment often proves more demanding than anticipated by the patients. Trying to support the patient throughout the course of treatment is therefore highlighted as important, for example, by seeing the patient more often or giving them more time in treatment.

The therapists discussed the importance of constantly respecting the enormous effort that treatment requires for the patients, and the disadvantages treatment can cause for the patient were emphasized. The therapists understood that the ED might be an important coping strategy for many patients, and that treatment entails giving up this coping strategy. Losing the ED as a coping mechanism can make it difficult for the patient to handle everyday life and continue treatment.

I also had a case with a person struggling with anorexia. She was making significant progress and had insightful reflections on what she had missed out on due to the extreme underweight and the excessive focus on extreme activities and orthorexic behaviours. Even though she had been dealing with it for a long time, I started to believe that she could overcome it. However, when she reached a normal BMI and began connecting with her emotions, it became overwhelming for her. She couldn’t handle those feelings, and her avoidant tendencies kicked in. She didn’t want to experience anxiety and despair, so she resorted to losing weight again. It was painful to witness. [«Yes»] She simply couldn’t manage it.

Respondent P, clinical psychologist, interview 5

In several of the group discussions the therapists talked about how letting go of the ED might cause intense fear of getting well, losing control, and making life changes that would make it difficult to stay in treatment for patients.

I think that it becomes too frightening, and too intense, and sort of life-changing to make the necessary changes. It’s like, ‘Oh, no, this was scary,’ and even though most people say, “Of course, I won’t have an eating disorder for the rest of my life,” right now it feels overwhelming in a way.

Respondent E, clinical psychologists, interview 2

Yes, I think so too. Often, there’s a fear of change. They may not have the strength to endure it.

Respondent G, clinical psychologists, interview 2

In the exploration of factors influencing premature terminations of treatment, therapists shed light on a challenge arising from the discordance between patients and clinicians concerning the definition of an optimal BMI. While the therapists put forward the necessity of maintaining a BMI above 18.5 for achieving and sustaining overall health, they experienced patients struggled to accept this. The therapists shared experiences where patients were resistant to the idea of increasing their BMI to values recommended by clinicians, such as 20 or higher. This disagreement often results in weight becoming a breaking point in treatment. The therapists address how many patients experience improvement when they gain weight but stagnate at a time when perceived costs are felt higher than the benefits of treatment.

In the early stages, before the treatment begins or early in the course of treatment, they experience a lot of symptoms that are difficult for them to endure. They often want to make a change. They are troubled by their feelings, and are unable to attend school, engage in leisure activities, or spend time with friends or family. Yes, there are many negative aspects of the eating disorder that a lot of people go through. But when they start to improve and regain some level of functioning, I get the impression that many think, “Well, I can live with the eating disorder to some extent and still live an okay life. I’m not willing to sacrifice more for complete recovery.” It seems like the pressure they’ve been under has eased, and it’s not as urgent for them to continue treatment. That’s my impression. The ambivalence, which is always present, begins to shift the other way again with the thought of “I don’t want to continue with this treatment program”.

Respondent P, clinical psychologist, interview 5

A specific example mentioned in one of the interviews is the «BMI-17 syndrome». This phenomenon is described as the tendency for some patients to quit treatment when they reach BMI 17. At this stage the patients begin to experience the positive consequences of weight gain. At the same time, however, the cost of treatment is still perceived as high, and patients drop out of treatment even though the risk of relapse is described as sky-high by the therapists. The therapists point out the importance of exploring this ambivalence together with patients. They use interventions where they highlight both the advantages and disadvantages of being ill and being able to get well. In one of the interviews, it was also discussed how a high BMI combined with a desire to lose weight can make treatment contraindicated.

Limited resources in the healthcare system, combined with a perception that it would be unethical to leave the patient in something so difficult for a long time, could lead to the therapist terminating treatment. Being in a long-lasting ambivalence exploration and/or if the patient struggled with severe comorbidity could be arguments for the therapists to end treatment.

Well, there’s an important ethical aspect to this, as we discussed earlier. It’s related to the time we have available. I believe that the time factor is crucial. For instance, if we give someone three years to recover from anorexia, they might become disillusioned over time. Having such a long timeframe can make it tough to endure the gradual changes. Making change is so hard for them (*the patients*), it would be ethically questionable to give them unlimited time. So, it’s about understanding what lies beneath and being clear that it’s perfectly okay to not have the strength to continue right now. However, I still believe that recovery is possible.

Respondent M, nurse, interview 4

## Discussion

The findings in this study suggest that most therapists accepted that dropout could be a part of a longer process towards recovery. Further, they conceptualized preparation for treatment as separate from treatment itself. In this phase the therapists gave information about treatment, assed comorbid symptoms and situational factors, and explored the patient’s willingness to engage in behavioural change. This preparation phase was seen as crucial for good treatment results by allowing the patients to actively choose treatment, addressing potential barriers to treatment, and preparing for obstacles and strains that are likely to occur during treatment of EDs. Consequently, the therapists would often invest much time and effort in this phase that could consist of several sessions with the patient and last for weeks, sometimes even months.

Interestingly, termination of treatment, either initiated by the patient or by the therapist, in the preparation phase of treatment was not necessarily perceived as dropout by the therapists. Leaving treatment at this stage was not referred to as something negative, as there was an understanding that this could lead to the patient returning at a later, more suitable time when ready. Hence, letting the patient leave therapy in the preparation phase would give the patient a better chance for re-entering and completing treatment later. Understanding of dropout in therapy as a potential positive outcome and a possibly necessary phase of a longer recovery process differs from much of the research literature, where dropout is associated with various negative and adverse consequences such as poorer treatment outcomes and prognosis [13]. This tension between existing research and the therapists’ conception of what constitutes dropout, and the meaning of dropout in our study is interesting and relates to current developments in the field. Seidinger-Leibovitz and colleagues have, for example, argued that a paradigm shift is necessary, where one moves away from a one-sided focus on negative patient characteristics predicting dropout [19]. One study even found that a high number of patients that dropout actually improves [30]. This points to the importance of understanding the processes of dropout and treatment completion better, including the relational dynamics in these processes.

The therapeutic alliance has been found to be one of the strongest predictors of outcome in psychotherapy research [31, 32] and is commonly conceptualized as consisting of agreement between therapist and patient on treatment goals and methods, as well as the bond between therapist and patient [33]. It is interesting to relate the findings in this study to the literature on therapeutic alliance, as it clarifies an inherent tension in the data, and between the three themes. As evident from theme 1 and 2, therapists really emphasized reaching agreement on goals and methods as a prerequisite for successful treatment– emphasizing the importance of the patient perspective in theme 1, while the therapist’s responsibility to assess if there is a good enough fit and agreement on goals and methods are emphasized in theme 2. Yet, these findings are in some ways at odds with much of the literature on the therapeutic alliance. Negotiating the therapeutic alliance is often described as a more open process in broader psychotherapy research. However, an important context in this study was the predetermined and non-negotiable goal of gaining weight above BMI of 18,5 when treating AN, and the relatively set tasks that patients had to engage in to achieve this goal. This is also reflected in theme 3, where this non-negotiable goal might be part of what makes treatment too challenging for the patient at that specific time. The reason why a BMI below 18.5 is considered non-negotiable [34] is that patients with a lower BMI is considered underweight and therefore also affected by symptoms of being underweight, which can contribute to maintaining the eating disorder [35]. With such a low BMI, the chance of relapses is very high, and it will be difficult to progress in the treatment process as the patient will be affected by the underweight symptoms (e.g., reduced cognitive and emotional functioning). Having non-negotiables in therapy creates an interesting tension between building an alliance with the patient and simultaneously encouraging their acceptance of evidence-based treatment components known to be essential for change [36, 37].

Discussions in the field about implementation of evidence-based treatments for EDs have highlighted therapist drift from the treatment protocols as a main obstacle for recovery from EDs [3]. Even though “flexibility within fidelity” is a core concept in psychological treatment [38], the question of what is considered the central elements in treatment and no-negotiables and what elements could be modified still needs to be handled by the therapists. The therapists interviewed in this study described investing much effort into preparing patients for considered necessary actions and defined goals, making them able to recover and stay well. The concept of being ‘ready for treatment’ was generally understood as being willing to make behavioural changes, which for most patients would involve regular eating, reducing vomiting, and/or gaining weight. Preparation for treatment in line with this involved making the rationale for the goals and methods understandable and acceptable for the patients, rather than freely negotiating them. Adherence to treatment protocols when making these preparations was described as important and consequently treatment might not be offered if it was not possible for the patient to agree on these predefined goals and methods.

It is interesting that “disagreement on treatment needs”, and “rigid standard procedures” is described as barriers for accepting treatment by patients [39]. Andersen et al. [39] found that from patients’ declining therapy perspectives, treatment was characterized by inflexible standard procedures that could not be adapted to their individual situations and preferences. The patients interviewed felt that the therapists did not listen to them, and they experienced a loss of identity, feeling reduced to an eating disorder rather than being seen as real people. The therapists in our study consistently emphasized that the decision to engage in treatment should ultimately be the patient’s decision, but at the same time they stressed the importance of adherence to treatment protocols. This tension between alliance-building and adherence to treatment protocols underscores the delicate balance clinicians face in promoting change while respecting patient autonomy. This balance might be more challenging in the treatment of EDs than some other mental illnesses because of the ego-syntonic character of the disorder [40, 41]. It is notable that the relationship between therapeutic alliance and treatment outcomes in ED treatment has been sparsely examined [42] and still remains uncertain [43, 44]. Further research will therefore be required to better understand how the therapeutic alliance influences ED treatment.

In our study therapists appear to feel responsibility for assisting patients in finding the right timing for treatment, ensuring that they do not deprive patients of future opportunities for recovery. This understanding of treatment means that some patients are not offered treatment despite their expressed desire for it. At the same time, there appears to be a shared understanding that allowing patients to make their own choices can enhance their engagement in treatment and reduce passivity. Some therapists pointed out that they deliberately used techniques where the patient had to take responsibility and actively choose their treatment to engage them and help them stay in the treatment process. This seems to create a tension between respecting patients’ choices and allowing them to decide for themselves, while also serving as a kind of “gatekeeper” who offers treatment only to patients they perceive as entirely ready for it. This perspective on treatment highlights a potential dilemma in the field: individuals might be prevented from starting treatment as their ‘golden ticket’ remains out of reach. In fact, some studies have identified “service restrictions (e.g., being ineligible to access a service due to strict entry criteria” as a potential treatment barrier in eating disorders [45]. This could be problematic since early intervention could improve outcomes and facilitate recovery for eating disorders [46].

It can be debated whether this way of understanding and discussing drop-out reflects a method-specific approach since all the therapists have received training in CBT-E. CBT-E training may influence therapists’ attitudes towards dropout by providing them with specific tools and strategies to manage and reduce dropout rates. The training emphasizes the importance of engagement and retention, which can shape therapists’ perceptions and approaches. In CBT-E there is a preparation phase whereby it is determined whether patients are in a situation that enables them to commit to the demands of treatment. Further research is needed to explore the direct impact of CBT-E training on therapists’ attitudes and patient outcomes. Additional studies would also be required to explore how therapists with different therapeutic approaches understand and work with dropout. When dropout is not adequately addressed, a significant number of individuals who would benefit from eating disorder treatment may not receive the necessary care. This can lead to worsened mental health outcomes and increased strain on healthcare resources.

Another key consideration within this discussion is access to resources within the health care system. Engaging patients, creating a secure therapeutic environment, exposure to food and addressing the emotional factors contributing to EDs takes both time and resources. With both being limited, therapists are forced to make difficult choices. They might need to prioritize the patients they believe would benefit the most from treatment or use less time per patient, or a combination of both, potentially compromising the access to and/or quality of treatment. It will, however, be important to explore empirically therapists’ conceptualization of dropout as a necessary phase of a longer recovery process and provide data that allows us to analyse whether patients who dropout eventually return to treatment and recover.

### Methodological reflexions

As in all other qualitative research, it will be difficult to generalize the results directly to the larger population of therapists within the field of ED. The findings may be unique to our particular groups or settings, and further research is required to explore transferability to other contexts. A strength of this study, however, is that it has given us the opportunity to examine therapists’ understanding of dropout in depth. To the best of our knowledge, there are few if any earlier studies on this topic. As this is an understudied area, we argue that it was beneficial to have an exploratory aim and design. Using FGIs, this study uncovers insights that would be challenging to detect through questionnaires or other quantitative methods. A potential limitation of this design, however, is that it is prone to potential group effects. For example, some informants may have made statements that did not reflect their own opinions and instead expressed themselves based on what they believed would be perceived as correct by the rest of the group. Perhaps some participants refrained from expressing their opinions in fear of being perceived as incompetent or controversial.

To minimize potential group effects, we held an introduction where we emphasized that there were no right or wrong answers at the beginning of the interviews. We assured participants that their responses would be treated confidentially. Additionally, we made efforts to include everyone by asking introductory questions and we tried to vary the questions, incorporating both open-ended questions that promoted discussion and individual questions that required personal responses. Furthermore, a moderator was present to facilitate the discussion and hopefully ensure that all participants had the opportunity to express their views.

During the analysis, we found that CBT-E training most likely has influenced how participants conceptualized and handled dropout. Therefore, it would have been beneficial to systematically collect information about the participants’ experiences and training prior to the interviews. This would have made it easier to integrate therapists’ formal training into the analysis and interpretation of data. Nevertheless, further research would be required to explore how therapists’ views and formal training affects the admission process and availability to treatment. Quantifying this impact requires systematic data collection and analysis, which was beyond the scope of this study but is an important area for future research.

Additionally, this study has uncovered new issues that could stimulate further research. Addressing how we can prevent patients from dropping out when the treatment becomes challenging could be an important issue for follow-up studies. It would be interesting to examine the balance between keeping patients engaged in treatment while simultaneously adhering to evidence-based and effective techniques, such as establishing regular eating patterns to gain weight. Furthermore, continued exploration of the tension this could create on the therapeutic alliance and how it affects treatment outcomes would be needed.

## Conclusion

The analysis of therapist perspectives on dropout in ED treatment indicates that termination of treatment was not necessarily perceived as dropout by the therapists. They emphasized the importance of offering treatment in a manner that is non-coercive, informative, respectful, and engaging to reduce dropout rates. Allowing the patient to choose therapy without feeling forced was seen as crucial for patient involvement in therapy.

At the same time, therapists highlighted the significance of assisting patients in finding the optimal timing for treatment when they are most likely to be receptive and able to participate successfully. Potential barriers in the patient’s life could lead therapists to withhold treatment. One reason for withholding treatment was the concept of a “one shot,” the understanding of a singular or limited opportunity for recovery. Therapists felt a responsibility to ensure they did not initiate treatment prematurely, thereby depriving the patient of the opportunity to recover.

Consequently, therapists may choose not to initiate treatment at the “wrong” time, despite patients’ willingness to, reserving the “golden ticket” for those with the best chance of success. Treatment was described as demanding as it would involve extensive behavioural change, letting go of the ED as a coping mechanism and fear for many of the patients. The therapists emphasized the importance of individual adaptations in treatment while simultaneously adhering to treatment protocols. Lack of progress or failure to meet treatment requirements, like weight gain, often led to treatment termination being initiated by the therapist.

This created an interesting tension in the data material: the therapists would stress the importance of patients’ self-autonomy and choice while simultaneously serving as “gatekeepers” only offering treatment for the patients they believed could attend treatment successfully. Due to the demanding nature of treatment the therapists were careful concerning who received treatment. Some of the therapists highlighted how limited resources within the health care system would mean that they had to prioritize the patients they believe would benefit the most from treatment, raising the bar for those who receive treatment even more.

There seemed to be a consensus that participating in treatment that does not progress could be more harmful than beneficial. Not being able to attend treatment, whether due to the patient’s choice to decline or discontinue, or because treatment was not offered, was not necessarily viewed negatively by therapists. Termination of treatment was recognized as a necessary evil for some patients as it could provide them a better opportunity to successfully attend treatment later at a more suitable time.

## Data Availability

Data sharing is not applicable to this article as it contains personally identifiable information, and individual privacy could be compromised. Data supporting the results is reported in the article.

## Appendix A

Focus group interview with therapists

(Information, introduction, and consent signing)

First of all, we want to thank you for participating and taking the time to help us gain a deeper understanding of dropouts from eating disorder treatment from your perspective as therapists. As you saw in the information sheet, this is part of a larger project where we also use registry data to examine predictors of dropout. Additionally, we are interviewing patients who have discontinued treatment for eating disorders to understand their experiences and perspectives. As you know, our conversation will be recorded for transcription purposes, but all material will be de-identified before publication. Do you have any questions before we begin?

1. Let’s start with some brief questions related to the context, so we have a better foundation for understanding your experiences. Can you first tell us how you typically work together? Are you part of a fixed treatment team, or do you work more individually? What kind of treatment is offered for patients with eating disorders here?
2. Great. Now that we have a clearer picture of the context you work in, let’s move on to the main theme of our conversation. You’re here because you have experience working with patients with eating disorders, and we know that many patients who start treatment for eating disorders end up dropping out. From your experience, how do you recognize a patient who is at risk of dropping out of treatment for eating disorders? What signs do you look for?
3. In your experience, what factors contribute to patients dropping out of treatment for eating disorders? (How do you understand this phenomenon? )
4. When you fear that a patient might drop out of treatment or assess that they are at risk, what strategies have you found effective? What challenges have you encountered?
5. What do you believe is necessary to keep more patients in treatment when they are at risk of dropping out of treatment for eating disorders?

## Abbreviations

AN: Anorexia nervosa
BED: Binge eating disorder
BN: Bulimia nervosa
CBT-E: Enhanced cognitive-behavioural therapy for eating disorders
ED: Eating disorders
ROS: Counselling on Eating Disorders (in Norwegian “Rådgivning om spiseforstyrrelser”, ROS)
RTA: Reflexive thematic analysis
